# Development and validation of a prediction model based on comorbidities to estimate the risk of in-hospital death in patients with COVID-19

**DOI:** 10.3389/fpubh.2023.1194349

**Published:** 2023-05-26

**Authors:** Yangjie Zhu, Boyang Yu, Kang Tang, Tongtong Liu, Dongjun Niu, Lulu Zhang

**Affiliations:** ^1^Department of Military Health Management, College of Health Service, Naval Medical University, Shanghai, China; ^2^Department of Medical Health Service, General Hospital of Northern Theater Command of PLA, Shenyang, China; ^3^Department of Medical Health Service, 969th Hospital of PLA Joint Logistics Support Forces, Hohhot, China

**Keywords:** COVID-19, comorbidity, hospitalization, death, prediction model, retrospective study

## Abstract

**Background:**

Most existing prognostic models of COVID-19 require imaging manifestations and laboratory results as predictors, which are only available in the post-hospitalization period. Therefore, we aimed to develop and validate a prognostic model to assess the in-hospital death risk in COVID-19 patients using routinely available predictors at hospital admission.

**Methods:**

We conducted a retrospective cohort study of patients with COVID-19 using the Healthcare Cost and Utilization Project State Inpatient Database in 2020. Patients hospitalized in Eastern United States (Florida, Michigan, Kentucky, and Maryland) were included in the training set, and those hospitalized in Western United States (Nevada) were included in the validation set. Discrimination, calibration, and clinical utility were evaluated to assess the model's performance.

**Results:**

A total of 17 954 in-hospital deaths occurred in the training set (*n* = 168 137), and 1,352 in-hospital deaths occurred in the validation set (*n* = 12 577). The final prediction model included 15 variables readily available at hospital admission, including age, sex, and 13 comorbidities. This prediction model showed moderate discrimination with an area under the curve (AUC) of 0.726 (95% confidence interval [CI]: 0.722—0.729) and good calibration (Brier score = 0.090, slope = 1, intercept = 0) in the training set; a similar predictive ability was observed in the validation set.

**Conclusion:**

An easy-to-use prognostic model based on predictors readily available at hospital admission was developed and validated for the early identification of COVID-19 patients with a high risk of in-hospital death. This model can be a clinical decision-support tool to triage patients and optimize resource allocation.

## Introduction

Since the first report of COVID-19 in late 2019, there have been approximately 756 million confirmed cases and over 6 million deaths worldwide (1). Surging COVID-19 hospital admissions pose enormous challenges for healthcare systems. To optimize limited resource allocation and prevent disease deterioration, clinicians need to identify patients with COVID-19 who are at high risk of death. Recent evidence has confirmed the association between abnormal imaging manifestations, biochemical results, demographic parameters, comorbidities, and adverse outcomes in patients with COVID-19 (2–4).

Based on the growing knowledge of the risk factors for severe COVID-19, risk stratification models incorporating multiple independent predictor variables have been developed to evaluate the risk of adverse outcomes and support patient management (5, 6). It is worth noting that unreliable prediction might do more harm than good in guiding medical decision-making (7). A systematic review found that most prognostic models for COVID-19 have a high risk of bias owing to inadequate sample sizes and unclear reports on model development and validation (8). Therefore, a prognostic model with a large representative dataset of hospitalized patients with COVID-19 is required for the complete evaluation of model performance.

Most COVID-19 patients have a mild clinical course, while some experienced rapid deterioration from the onset of symptoms into severe illness requiring hospital admission (3, 9). Within patients in need of escalated clinical care, rapid identification of those with high risk of poor outcomes at hospital admission will facilitate appropriate supportive care, avoid disease progression, and alleviate the burden on the health system. However, existing models often include predictor variables, such as imaging manifestations and laboratory results (10, 11), which cannot be acquired at hospital admission. These clinical parameters limit the application of prognostic models for the early identification of high-risk patients, especially when hospitals face a massive influx of patients with COVID-19.

We aimed to develop and validate an easy-to-use prognostic model that uses demographic and comorbidity data routinely available at hospital admission to predict in-hospital death in hospitalized patients with COVID-19.

## Methods

### Study design and participants

In this retrospective cohort study, we included hospitalized patients admitted for the diagnosis of COVID-19 using the Healthcare Cost and Utilization Project (HCUP) State Inpatients Database (SID) of Nevada, Florida, Michigan, Maryland, and Kentucky in 2020. The HCUP SID is a longitudinal database containing the inpatient discharge records of state hospitals. The requirement for informed consent was waived because the HCUP SID was de-identified. All HCUP data users completed an HCUP Data Use Agreement. Model development, validation and reporting followed the guidelines of the Transparent Reporting of a Multivariable Prediction Model for Individual Prediction or Diagnosis (TRIPOD) (12). This study was approved by the Ethics Committee of the Naval Medical University (No. 2021LL024).

**Figure 1:**
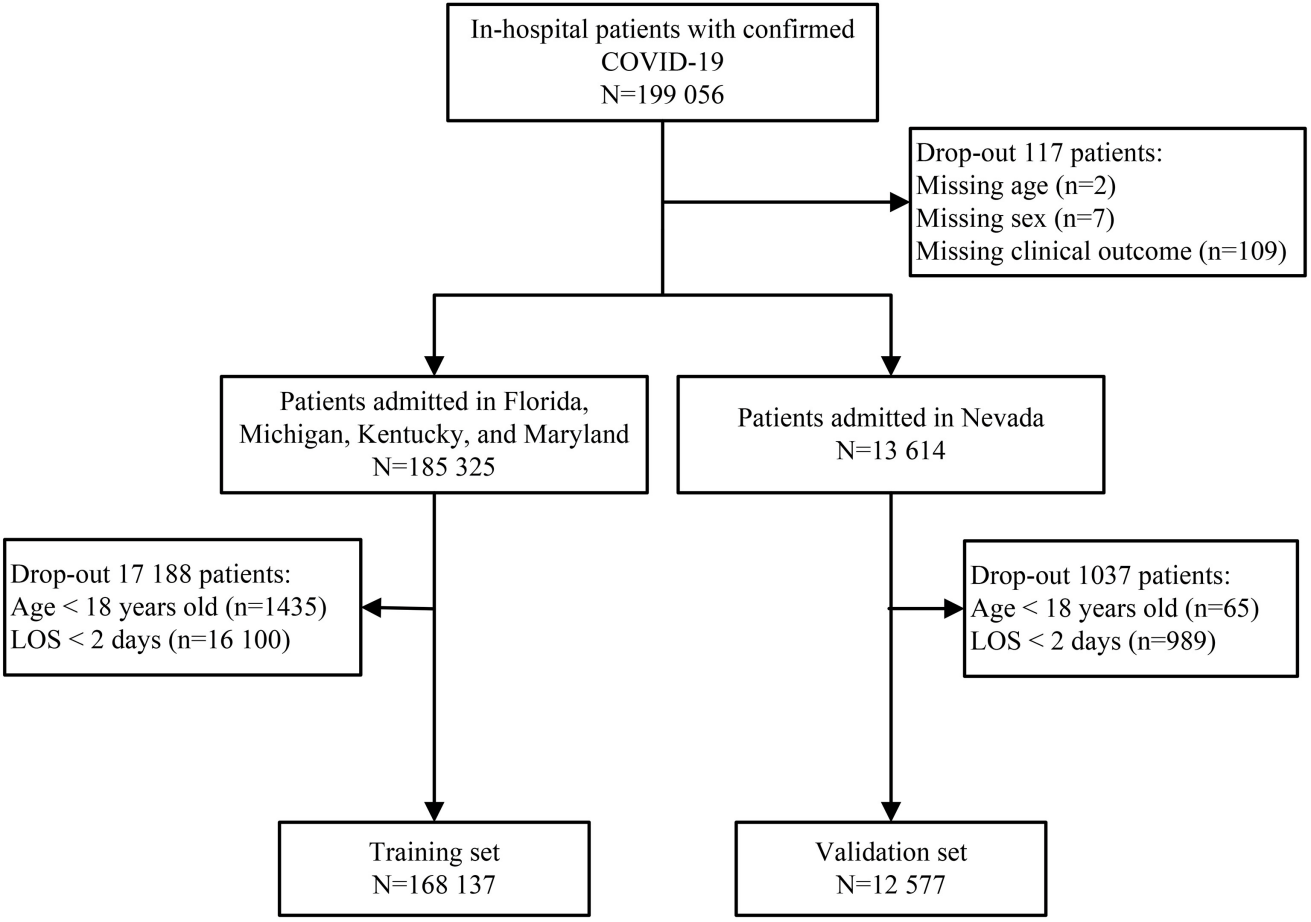
Flow chart of study participants in the train and validation set.

Patients hospitalized with an admitting diagnosis of COVID-19 were included in this study. The COVID-19 diagnosis was identified according to the International Classification of Disease 10th revision (ICD-10) code (U071) (13). Patients aged < 18 years or those with a length of stay < 48 h, were excluded. Missing data were observed for three variables: age (2 patients), sex (7 patients), and clinical outcome (109 patients) (Figure 1). These records were excluded because the percentage of missing values was < 0.06% (117/199 056).

### Outcomes

The primary outcome was in-hospital death of patients with COVID-19. This outcome was selected because early identification of patients at a high risk of adverse outcomes can support clinical decision-making to decrease COVID-19-related mortality.

### Predictor variables

Based on a review of the existing literature to identify factors associated with COVID-19 mortality, the following predictors available at hospital admission were selected to predict in-hospital mortality: age, sex, and Elixhauser Comorbidity Index (ECI) (14, 15). The ECI identified a set of preexisting conditions that were unrelated to the principal diagnosis and significantly impacted resource allocation and in-hospital mortality (16). A total of 38 comorbidities were included in the HCUP Elixhauser Comorbidity Software tool (version 2022.1), which was originally developed using the International Classification of Diseases, Tenth Revision, Clinical Modification (ICD-10-CM). As the primary intention was to develop an easy-to-use prediction model for bedside use, age was converted into four categories (≤ 60, 60–69, 70–79, or ≥ 80 years) in the final model.

### Model development

Patients hospitalized in Eastern United States (Florida, Michigan, Kentucky, and Maryland) in 2020 were included in the training set. Considering the rule of at least 10 events per candidate predictor parameter (10 EPP) (17), a sample size of 17,954 in-hospital deaths in the training set was sufficient for 40 predictor variables.

Using 10-fold cross validation, we performed a least absolute shrinkage and selection operator (LASSO) logistic regression for predictor selection to minimize overfitting and potential collinearity of variables (18). LASSO logistic regression was conducted to fit the prediction model for all lambda and to use the one standard error (1SE) rule to select the lambda, which can reduce dimensionality and optimize the prediction model. Variables selected by LASSO logistic regression were subsequently assessed using a standard binary logistic regression (enter method). Finally, a prognostic nomogram for predicting the risk of in-hospital death in patients with COVID-19 was constructed based standard binary logistic regression results.

Discrimination of the prediction model was evaluated using the area under the curve (AUC) of the receiver operator characteristic (ROC). The Youden index was used to determine the optimal cutoff point that maximized sensitivity and specificity. Overall goodness of fit was assessed using the Brier score. A calibration curve was used to examine the agreement between the predicted and observed in-hospital deaths. Decision curve analysis (DCA) was performed to validate clinical utility by calculating the net benefits at different threshold probabilities.

To evaluate the discriminatory performance of the prediction model in different settings, we conducted a sensitivity analysis with complete data that included patients aged < 18 years old or hospitalized for < 2 days. Given the reported association between ethnicity and COVID-19-related death, further sensitivity analysis was performed by stratifying the training and validation sets by ethnicity (19).

### Model validation

Patients hospitalized in Western United States (Nevada) in 2020 were included in the validation set. The prediction model's performance was assessed based on discrimination, calibration, and clinical utility. A sensitivity analysis was also performed with the complete data and stratification by ethnicity of the validation set.

### Statistical analysis

Continuous variables were presented as mean (standard deviation, SD) or median (interquartile range, IQR), and categorical variables as frequencies (%). The demographic and clinical characteristics of the patients were analyzed using the Kruskal–Wallis test, Chi-square test, or Fisher's exact test, as appropriate. The standard binary logistic regression results are reported as coefficients and odds ratios (OR) with 95% confidence intervals (CI). All tests were two-sided, and statistical significance was set at *p* < 0.05. All statistical analyses were performed using R software (version 4.0.3). The packages used in this study included “glmnet”, “rms”, “pROC”, “calibrate”, “dca.R”, and “lrm”.

## Results

### Baseline characteristics

A total of 199 056 patients with COVID-19 were screened for eligibility, and 180 714 patients were assigned to the training set (*n* = 168 137) and validation set (*n* = 12 577) (Figure 1). The median age was 67 years (IQR, 55—78), ranging from 18 to 109 years old. There were 61 795 (34.19%) patients aged < 60 years old, 40 024 (22.15%) patients aged 60—69 years old, 40 260 (22.28%) patients aged 70—79 years old, and 38 635 (21.38%) patients aged ≥ 80 years old. Of these patients, 84 970 (47.02%) were female, 89 052 (49.28%) were of white ethnicity, and 87 235 (48.27%) were of non-white ethnicity. There were 73 076 (40.44%) patients with over three comorbidities and 19 306 (10.68%) deaths during hospitalization. In the training set, the mortality was 11.06% (8683/78 498) in the white ethnic group and 10.17% (8667/85 212) in the non-white ethnic group. Similar results were observed in the validation set, with 11.24% (1186/10 554) mortality in the white ethnic group and 8.21% (166/2023) mortality in the non-white ethnic group.

The most common comorbidity was uncomplicated hypertension (77 656, 42.97%). Other comorbidities such as obesity, complicated hypertension, diabetes with chronic complications, and chronic pulmonary disease, also affected more than 20% patients with COVID-19. Compared to surviving patients, those who died in the hospital were more likely to be male, older adults, and have more comorbidities. The baseline characteristics of the training and validation sets were presented in Table 1.

**Table 1:** Demographic and clinical characteristics for training and validation set of patients admitted to hospital with COVID-19.

**Characteristics**	**Training set**				**Validation set**			
	**Overall (n**=**168,137)**	**Survived** **(n**=**150,183)**	**Died (n**=**17,954)**	**P-value**	**Overall (n**=**12,577)**	**Survived** **(n**=**11,225)**	**Died (n**=**1,352)**	**P-value**
**Age (median, IQR)**	66 (54–78)	65 (53–77)	76 (66–84)	< 0.001	69 (58–78)	67 (57–77)	76 (68–84)	< 0.001
< 60 y	58,265 (34.65)	55,914 (37.23)	2,351 (13.09)		3,530 (28.07)	3,412 (30.40)	118 (8.73)	
60–69 y	37,022 (22.02)	33,458 (22.28)	3,564 (19.85)		3,002 (23.87)	2,731 (24.33)	271 (20.04)	
70–79 y	36,988 (22.00)	31,636 (21.06)	5,352 (29.81)		3,272 (26.02)	2,836 (25.27)	436 (32.25)	
≥80 y	35,862 (21.33)	29,175 (19.43)	6,687 (37.25)		2,773 (22.05)	2,246 (20.01)	527 (38.98)	
**Female**	78,627 (46.76)	71,327 (47.49)	7,300 (40.66)	< 0.001	6,343 (50.43)	5,718 (50.94)	625 (46.23)	0.001
**Ethnicity**				< 0.001				< 0.001
White	78,498 (46.69)	69,815 (46.49)	8,683 (48.36)		10,554 (83.92)	9,368 (83.46)	1,186 (87.72)	
Non-white	85,212 (50.68)	7,6545 (50.97)	8,667 (48.27)		2,023 (16.08)	1,857 (16.54)	166 (12.28)	
Not recorded	4,427 (2.63)	3,823 (2.55)	604 (3.36)		–	–	–	
**Number of comorbidities**				< 0.001				< 0.001
≤ 3	100,800 (59.95)	93,949 (62.56)	6,851 (38.16)		6,838 (54.37)	6,354 (56.61)	484 (35.80)	
> 3	67,337 (40.05)	56,234 (37.44)	11,103 (61.84)		5,739 (45.63)	4,871 (43.39)	868 (64.20)	
**AIDS**	1,354 (0.81)	1,227 (0.82)	127 (0.71)	0.131	34 (0.27)	33 (0.29)	1 (0.07)	0.232
**Alcohol abuse**	2,843 (1.69)	2,543 (1.69)	300 (1.67)	0.85	133 (1.06)	119 (1.06)	14 (1.04)	1
**Deficiency anemias**	33,480 (19.91)	28,081 (18.70)	5,399 (30.07)	< 0.001	1,921 (15.27)	1,647 (14.67)	274 (20.27)	< 0.001
**Arthropathies**	5,078 (3.02)	4,470 (2.98)	608 (3.39)	0.003	459 (3.65)	410 (3.65)	49 (3.62)	1
**Chronic blood loss anemia**	558 (0.33)	462 (0.31)	96 (0.53)	< 0.001	31 (0.25)	25 (0.22)	6 (0.44)	0.208
**Leukemia**	1,006 (0.60)	825 (0.55)	181 (1.01)	< 0.001	91 (0.72)	71 (0.63)	20 (1.48)	0.001
**Lymphoma**	1,153 (0.69)	929 (0.62)	224 (1.25)	< 0.001	88 (0.70)	76 (0.68)	12 (0.89)	0.481
**Metastatic cancer**	1,379 (0.82)	1,100 (0.73)	279 (1.55)	< 0.001	101 (0.80)	77 (0.69)	24 (1.78)	< 0.001
**Solid tumor without metastasis**, ***in situ***	20 (0.01)	18 (0.01)	2 (0.01)	1	2 (0.02)	1 (0.01)	1 (0.07)	0.515
**Solid tumor without metastasis, malignant**	2,910 (1.73)	2,470 (1.64)	440 (2.45)	< 0.001	201 (1.60)	165 (1.47)	36 (2.66)	0.001
**Cerebrovascular disease**	6,061 (3.60)	5,060 (3.37)	1,001 (5.58)	< 0.001	454 (3.61)	384 (3.42)	70 (5.18)	0.001
**Congestive heart failure**	25,195 (14.98)	20,236 (13.47)	4,959 (27.62)	< 0.001	2,548 (20.26)	2,106 (18.76)	442 (32.69)	< 0.001
**Coagulopathy**	16,285 (9.69)	13,459 (8.96)	2,826 (15.74)	< 0.001	957 (7.61)	776 (6.91)	181 (13.39)	< 0.001
**Dementia**	22,382 (13.31)	18,401 (12.25)	3,981 (22.17)	< 0.001	1,701 (13.52)	1,360 (12.12)	341 (25.22)	< 0.001
**Depression**	17,361 (10.33)	15,487 (10.31)	1,874 (10.44)	0.61	1,967 (15.64)	1,782 (15.88)	185 (13.68)	0.04
**Diabetes with chronic complications**	43,005 (25.58)	36,357 (24.21)	6,648 (37.03)	< 0.001	3,535 (28.11)	3,080 (27.44)	455 (33.65)	< 0.001
**Diabetes without chronic complications**	23,676 (14.08)	21,586 (14.37)	2,090 (11.64)	< 0.001	1,909 (15.18)	1,743 (15.53)	166 (12.28)	0.002
**Drug abuse**	2,434 (1.45)	2,267 (1.51)	167 (0.93)	< 0.001	201 (1.60)	188 (1.67)	13 (0.96)	0.063
**Hypertension, complicated**	44,913 (26.71)	36,747 (24.47)	8,166 (45.48)	< 0.001	3,902 (31.02)	3,288 (29.29)	614 (45.41)	< 0.001
**Hypertension, uncomplicated**	72,260 (42.98)	65,921 (43.89)	6,339 (35.31)	< 0.001	5,396 (42.90)	4,930 (43.92)	466 (34.47)	< 0.001
**Liver disease, mild**	7,077 (4.21)	6,395 (4.26)	682 (3.80)	0.004	487 (3.87)	443 (3.95)	44 (3.25)	0.241
**Liver disease, moderate to severe**	886 (0.53)	707 (0.47)	179 (1.00)	< 0.001	63 (0.50)	45 (0.40)	18 (1.33)	< 0.001
**Chronic pulmonary disease**	39,610 (23.56)	34,645 (23.07)	4,965 (27.65)	< 0.001	3,828 (30.44)	3,363 (29.96)	465 (34.39)	0.001
**Neurological disorders affecting movement**	4,437 (2.64)	3,825 (2.55)	612 (3.41)	< 0.001	541 (4.30)	468 (4.17)	73 (5.40)	0.042
**Neurological disorders unaffecting movement**	14,308 (8.51)	11,372 (7.57)	2,936 (16.35)	< 0.001	1,093 (8.69)	859 (7.65)	234 (17.31)	< 0.001
**Seizures and epilepsy**	6,103 (3.63)	5,332 (3.55)	771 (4.29)	< 0.001	512 (4.07)	447 (3.98)	65 (4.81)	0.168
**Obesity**	47,591 (28.30)	42,797 (28.50)	4,794 (26.70)	< 0.001	3,453 (27.45)	3,133 (27.91)	320 (23.67)	0.001
**Paralysis**	5,337 (3.17)	4,504 (3.00)	833 (4.64)	< 0.001	448 (3.56)	376 (3.35)	72 (5.33)	< 0.001
**Peripheral vascular disease**	6,926 (4.12)	5,662 (3.77)	1,264 (7.04)	< 0.001	515 (4.09)	415 (3.70)	100 (7.40)	< 0.001
**Psychoses**	7,432 (4.42)	6,714 (4.47)	718 (4.00)	0.004	502 (3.99)	450 (4.01)	52 (3.85)	0.83
**Pulmonary circulation disease**	4,470 (2.66)	3,587 (2.39)	883 (4.92)	< 0.001	323 (2.57)	273 (2.43)	50 (3.70)	0.007
**Renal failure, moderate**	21,291 (12.66)	17,299 (11.52)	3,992 (22.23)	< 0.001	2,040 (16.22)	1,735 (15.46)	305 (22.56)	< 0.001
**Renal failure, severe**	10,230 (6.08)	8,167 (5.44)	2,063 (11.49)	< 0.001	714 (5.68)	579 (5.16)	135 (9.99)	< 0.001
**Hypothyroidism**	23,093 (13.73)	20,171 (13.43)	2,922 (16.27)	< 0.001	2,346 (18.65)	2,064 (18.39)	282 (20.86)	0.03
**Other thyroid disorders**	2,124 (1.26)	1,942 (1.29)	182 (1.01)	0.002	127 (1.01)	116 (1.03)	11 (0.81)	0.535
**Peptic ulcer with bleeding**	790 (0.47)	673 (0.45)	117 (0.65)	< 0.001	50 (0.40)	38 (0.34)	12 (0.89)	0.005
**Valvular disease**	7,708 (4.58)	6,416 (4.27)	1,292 (7.20)	< 0.001	558 (4.44)	484 (4.31)	74 (5.47)	0.059
**Weight loss**	9,629 (5.73)	7,500 (4.99)	2,129 (11.86)	< 0.001	658 (5.23)	506 (4.51)	152 (11.24)	< 0.001

IQR, interquartile range; AIDS, Acquired immune deficiency syndrome.

### Model construction and performance assessment

**Figure 2:**
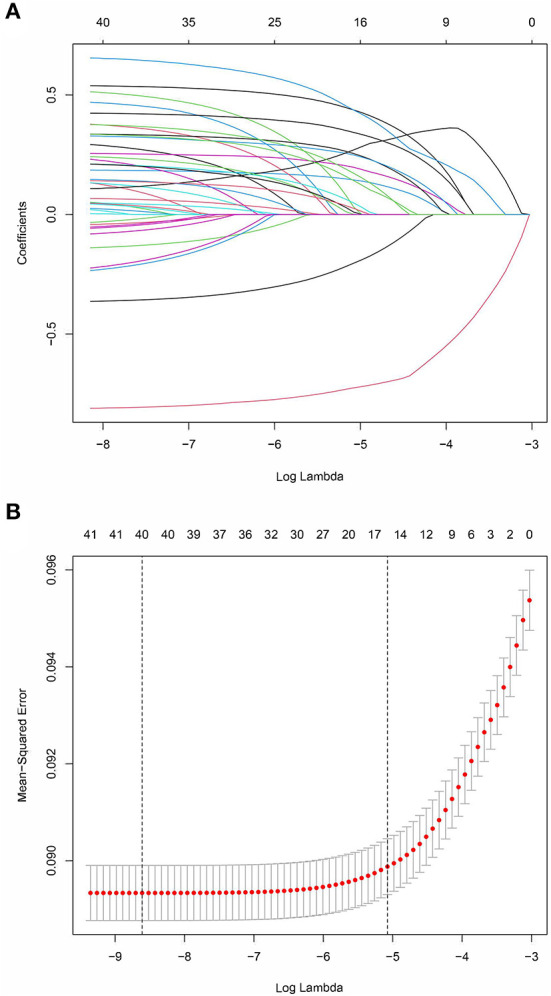
LASSO coefficient profiles of 40 candidate predictors **(A)** and 15 predictors selected using LASSO regression **(B)**.

**Figure 3:**
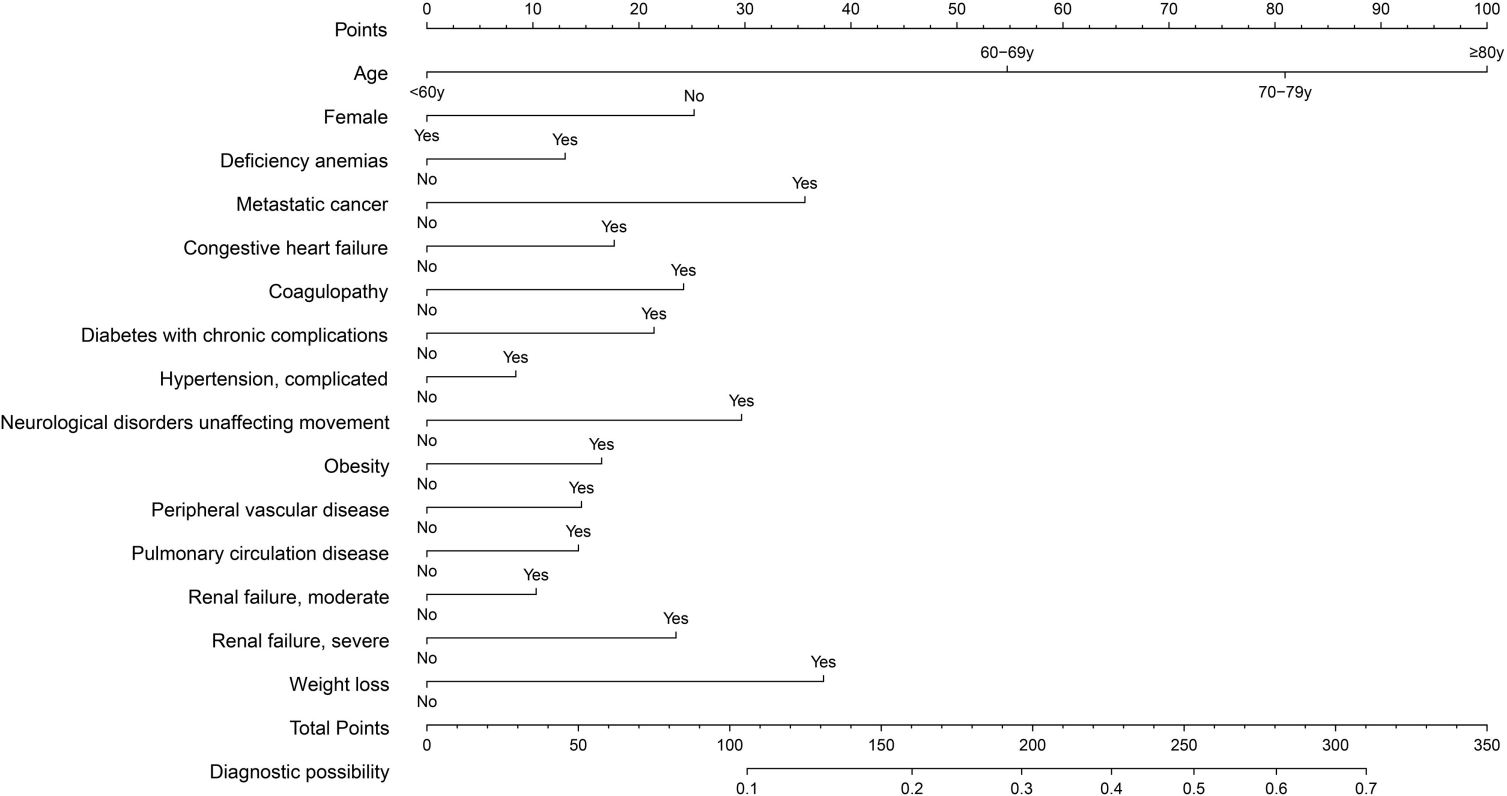
Nomogram for predicting in-hospital death in patients with COVID-19. Different values of each variable correspond to different positions in the nomogram. Draw a line from the position of each variable to the points axis for acquiring points of this variable. Points of different variables are calculated and summed to yield a total score that can be converted into predicted probability of in-hospital death.

The prediction model was developed using a training set of 168 137 patients, of which 17 954 in-hospital deaths occurred. In total, 40 candidate variables were screened using LASSO regression, and 15 independent variables were selected for multivariate logistic regression (Figure 2). In-hospital deaths increased with age, male sex, and 13 comorbidities: deficiency anemias, metastatic cancer, congestive heart failure, coagulopathy, diabetes with chronic complications, complicated hypertension, neurological disorders unaffecting movement, obesity, peripheral vascular disease, pulmonary circulation disease, moderate renal failure, severe renal failure, and weight loss. Among these predictors, ORs were highest for those 60—69 years old (OR 2.26, 95% CI: 2.14—2.39), 70—79 years old (OR 3.34, 95% CI: 3.17—3.53), and ≥ 80 years old (OR 4.44, 95% CI: 4.2—4.69) (Table 2). Finally, a nomogram was generated by assigning a weighted score to each selected variable (Figure 3). The calculation of risk score was as follows: risk score = 55 (60–69 years old) + 81 (70—79 years old) + 100 (≥ 80 years old) + 25 (male) + 13 (deficiency anemias) + 36 (metastatic cancer) + 18 (congestive heart failure) + 24 (coagulopathy) + 21 (diabetes with chronic complications) + 8 (hypertension, complicated) + 30 (neurological disorders unaffecting movement) + 16 (obesity) + 15 (peripheral vascular disease) + 14 (pulmonary circulation disease) + 10 (renal failure, moderate) + 23 (renal failure, severe) + 37 (weight loss). The optimal cut-point was 107 (corresponding to a threshold probability 0.102), which divided patients into low-risk group (score ≤ 107) and high-risk group (score > 107).

**Table 2:** Multivariable logistic regression of risk factors for in-hospital death in patients with COVID-19 in the training set.

	**β**	**OR**	**95% CI**	**P-value**
Intercept	−3.4			
Age (years)				< 0.001
< 60	Reference	Reference	Reference	
60–69	0.82	2.26	2.14–2.39	
70–79	1.21	3.34	3.17–3.52	
≥80	1.49	4.44	4.2–4.69	
Female	−0.38	0.69	0.66–0.71	< 0.001
Deficiency anemias	0.19	1.21	1.17–1.26	< 0.001
Metastatic cancer	0.53	1.7	1.48–1.95	< 0.001
Congestive heart failure	0.26	1.3	1.24–1.37	< 0.001
Coagulopathy	0.36	1.43	1.37–1.5	< 0.001
Diabetes with chronic complications	0.32	1.38	1.33–1.43	< 0.001
Hypertension, complicated	0.13	1.13	1.07–1.2	< 0.001
Neurological disorders unaffecting movement	0.44	1.56	1.48–1.63	< 0.001
Obesity	0.25	1.28	1.23–1.33	< 0.001
Peripheral vascular disease	0.22	1.24	1.16–1.33	< 0.001
Pulmonary circulation disease	0.21	1.24	1.14–1.34	< 0.001
Renal failure, moderate	0.15	1.17	1.1–1.23	< 0.001
Renal failure, severe	0.35	1.42	1.33–1.52	< 0.001
Weight loss	0.56	1.75	1.66–1.84	< 0.001

OR, odds ratio; CI, confidence interval.

**Figure 4:**
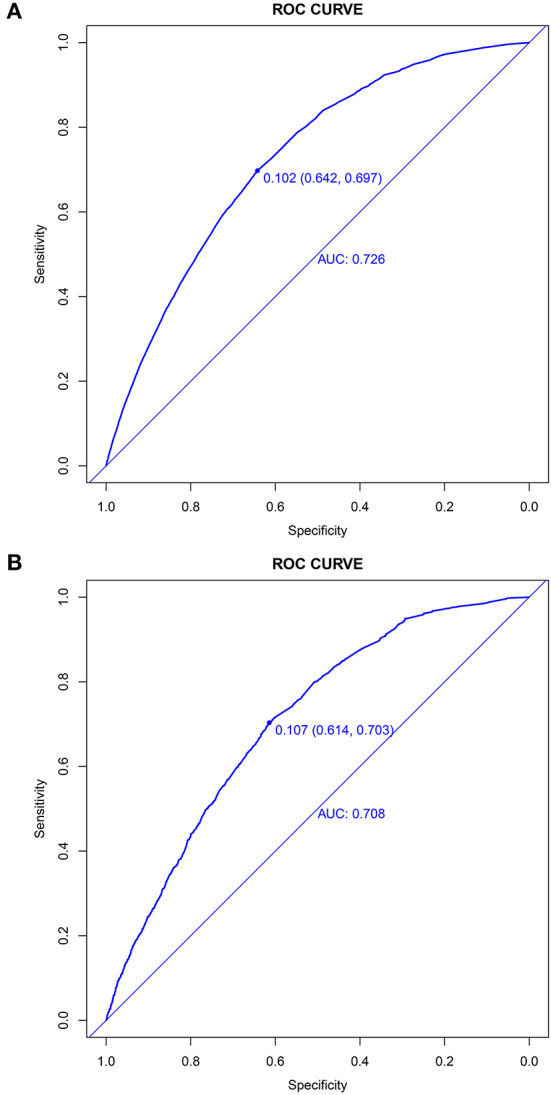
Discrimination of the nomogram for predicting in-hospital death in patients with COVID-19. Receiver operator characteristic curves of the nomogram in the training set **(A)** and validation set **(B)**.

**Figure 5:**
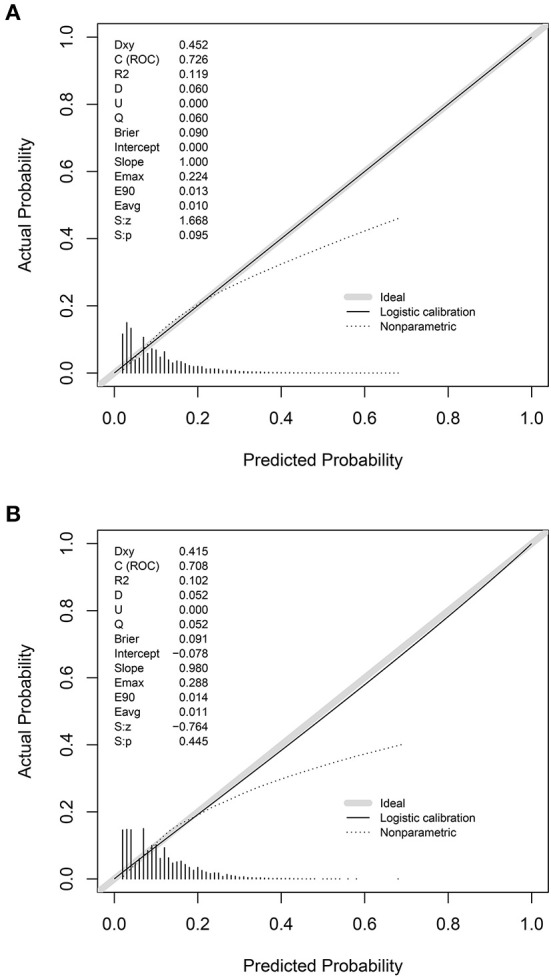
Calibration of the nomogram for predicting in-hospital death in patients with COVID-19. Calibration curves of the nomogram in the training set **(A)** and validation set **(B)**.

**Figure 6:**
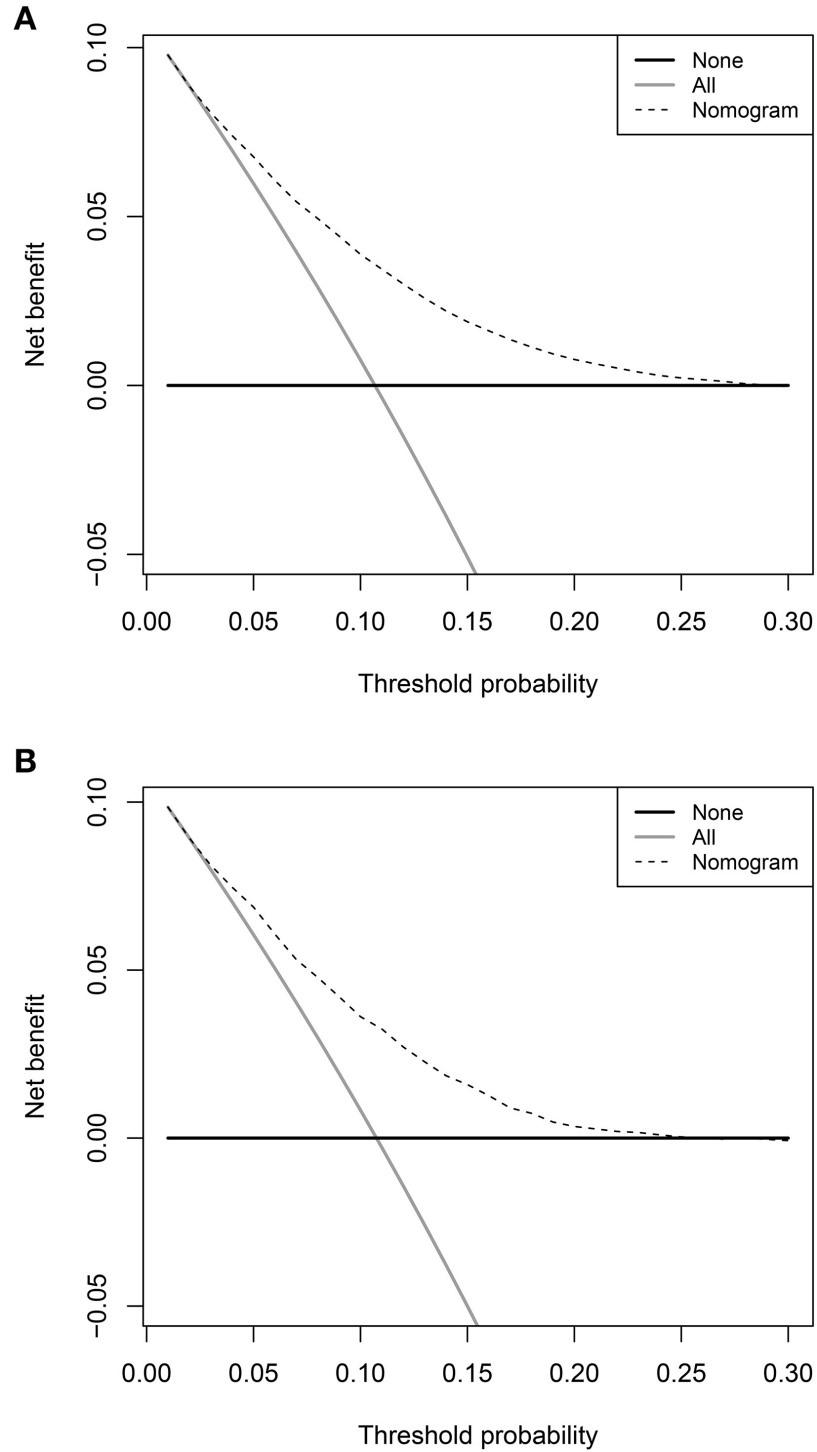
Decision curve analyses depicting the net benefits of the nomogram. The decision curve analyses of the nomogram in the training set **(A)** and validation set **(B)**.

The prediction model showed moderate discrimination for in-hospital deaths with an AUC of 0.726 (95% CI: 0.722—0.729) in the training set. Maximal discrimination was achieved at a cutoff value of 0.102 which provided a sensitivity of 0.642 and a specificity of 0.697 (Figure 4A). The calibration curve showed a slightly overestimated risk of in-hospital death in the training set (Brier score = 0.090, slope = 1, intercept = 0) (Figure 5A). DCA was conducted to assess the clinical utility of the present model (Figure 6A). When the threshold probability ranged between 3% and 29%, applying the nomogram to determine whether to add an intervention obtained a greater net benefit than treating no patients or all patients.

### Validation of the nomogram

The validation set consisted of 12 577 patients with 1352 in-hospital deaths. The AUC of the prediction model in the validation set was 0.708 (95% CI: 0.694—0.721), indicating moderate discrimination (Figure 4B). A cutoff value of 0.107 (sensitivity = 0.614, specificity = 0.703) was found to be the optimal level for discriminating in-hospital deaths in the validation set. The calibration curve also demonstrated a slightly overestimated risk in the validation set (Brier score = 0.091, slope = 0.98, intercept = −0.078) (Figure 5B). The DCA showed that when the net benefit of the nomogram was higher than the strategy of “treat all” and “treat none”, the threshold probability ranged between 3% and 26% (Figure 6B).

### Sensitivity analysis

Sensitivity analysis was conducted using complete case data, which included patients aged < 18 years old or hospitalized for < 2 days. The prediction model showed similar discrimination with an AUC of 0.733 (95% CI: 0.729–0.736) in the training set (*n* = 185 325) and an AUC of 0.711 (95% CI: 0.699–0.724) in the validation set (*n* = 13 614). Additionally, the prediction model showed better discrimination in the non-white ethnic group (AUC 0.774, 95% CI: 0.743–0.805) than the white ethnic group (AUC 0.697, 95% CI: 0.683–0.711) in the validation set. Similar results were observed in the training set: an AUC of 0.746 (95% CI: 0.740–0.751) in the non-white ethnic group and 0.708 (95% CI: 0.702–0.713) in the white ethnic group.

## Discussion

We developed and validated a prognostic model with 15 variables to predict in-hospital mortality in hospitalized patients with COVID-19 (*n* = 180,714). This prognostic model, based on age, sex, and comorbidities commonly available at hospital admission, performed well in terms of discrimination, calibration, and clinical utility. Moreover, sensitivity analyses showed moderate-to-good discrimination in different settings, confirming the robustness of our findings.

The present prediction model included predictors reflecting the patients' demographics and preexisting conditions, which are common components of other risk stratification models (20, 21). In our study, the in-hospital death risk showed a significant age gradient, supporting age as a strong predictor of mortality in hospitalized patients with COVID-19 (22). The increasing trend in mortality risk with age also suggested the necessity for early treatment intervention and priority resource allocation for older patients (23).

Comorbidities are usually presented in two forms in COVID-19 prognostic models: individual comorbidity indicators (24, 25) and an unweighted count of comorbidities (26, 27). In this study, the two most significant predictors of mortality among comorbidities were metastatic cancer and weight loss. They had significantly higher ORs than the other comorbidities. Therefore, these comorbidities were presented as individual features in the final prediction model. A previous study also confirmed that the prediction model for in-hospital mortality using individual comorbidity indicators had better discrimination than that including comorbidities given an equal weight (16).

The mortality rate for hospitalized patients in white ethnic group was higher than its non-white counterpart, and it was consistent with the results of a previous study (28). The disparity in COVID-19 mortality between different ethnic groups may result from the higher prevalence of most comorbidities in white hospitalized patients (29). Besides, white hospitalized patients (median age 72 years, IQR: 60–81) were significantly older than non-white patients (median age 62 years, IQR: 50–73). Comorbidities and disease severity tended to be associated with older age, and thus the white ethnic group with more older patients was more likely to develop comorbidities and have poor outcomes (30, 31).

A living systematic review identified 606 prognostic models of COVID-19 with a median C index of 0.81. However, a high risk of bias was observed in the majority (545/606) of these existing models (8). An unreliable prognostic prediction could be more harmful than beneficial in guiding clinical practice. The most common reason for the risk of bias was problematic methodology. Insufficient sample size and the number of events increased the risk of overfitting, and poor reports of model derivation and validation might lead to optimistic performance statistics (32). This study was conducted with a large sample size and strictly followed the recommendations of TRIPOD during all stages of design, implementation, and reporting, which was beneficial to reduce risk of bias.

The present prognostic model showed a moderate discriminatory performance, which was lower than that of another prognostic model (AUC 0.89) with a relatively small sample size (384 deaths in 2,492 patients) based on age, sex, and comorbidities (lymphoma/leukemia, liver disease, ischemic heart disease, dementia, chronic obstructive pulmonary disease, diabetes, and chronic kidney disease) (33). However, we could not to validate its performance in our cohort because ischemic heart disease was not included in the 38 comorbidity measures of the HCUP SID.

This study had several strengths. Using a large inpatient database with an event-to-parameter ratio >400 reduced the risk of overfitting in the prediction model (34). As a large number of candidate risk predictors might cause inaccurate estimations with multicollinearity, we performed LASSO regression to minimize potential multicollinearity. In addition, using predictor variables commonly available at the time of hospital admission strengthened its clinical applicability for the early identification of high-risk patients, avoiding the requirement for imaging manifestations and laboratory results that were only available in the post-hospitalization period. Moreover, sensitivity analyses were conducted to confirm the robustness of the model performance.

Our study had some limitations. First, records with missing values were excluded, and complete data analysis may have caused a loss of precision and power (35). Considering the substantially small proportion of missing values that mainly existed in the outcome variable (109/117), we excluded missing data instead of applying multiple imputation methods. Second, some rare comorbidities were not recorded in the HCUP SID group; including these comorbidities might have improved our prognostic model's performance. However, the large number of comorbidities identified by the ECI made it impossible to neglect important comorbidities with large effects on in-hospital mortality. Finally, the present model derived from a database only including patients limited to a certain area in the USA in 2020 might limit its generalization in the current clinical practice as the performance of our model was likely to vary over time and differ between regions with the emergence of new COVID-19 variants and imbalanced regional vaccination (36–38). Further validation using large representative datasets from different time periods and geographical regions is required to confirm the applicability of the prognostic model.

## Conclusion

An easy-to-use prediction model incorporating age, sex, and comorbidities was developed to evaluate the risk of in-hospital death in patients with COVID-19. Using predictors routinely acquired at hospital admission enables early identification of high-risk patients. This simple risk-stratification model can support clinical decision-making to guide patient management and optimize resource allocation when facing a surging number of infections.

## Data availability statement

The raw data supporting the conclusions of this article will be made available by the authors, without undue reservation.

## Ethics statement

The studies involving human participants were reviewed and approved by the Ethics Committee of the Naval Medical University. The Ethics Committee waived the requirement of written informed consent for participation.

## Author contributions

YJZ, BYY, KT contributed to study design, data acquisition, statistical analysis, and manuscript preparation. TTL contributed to study design and data acquisition. DJN contributed to data acquisition and manuscript preparation. LLZ contributed to the study conception, design, data interpretation, manuscript editing, and funding acquisition. All authors read and approved the final manuscript.

## References

[1] WHO. Coronavirus (COVID-19) Dashboard: Word Health Organization. (2023) Available onnline at: https://www//COVID19.who.int (accessed March 26, 2023).

[2] Xiong Y Sun D Liu Y Fan Y Zhao L Li X. Clinical and high-resolution ct features of the COVID-19 infection: comparison of the initial and follow-up Changes. Invest Radiol. (2020) 55:332–9. 10.1097/RLI.0000000000000674 32134800 PMC7147282

[3] Miyashita K Hozumi H Furuhashi K Nakatani E Inoue Y Yasui H. Changes in the characteristics and outcomes of COVID-19 patients from the early pandemic to the delta variant epidemic: a nationwide population-based study. Emerg Microbes Infect. (2023) 12:2155250. 10.1080/22221751.2022.2155250 36469641 PMC9788709

[4] Wadhera RK Wadhera P Gaba P Figueroa JF Joynt Maddox KE Yeh RW. Variation in COVID-19 hospitalizations and deaths across new york city boroughs. JAMA. (2020) 323:2192–5. 10.1001/jama.2020.7197 32347898 PMC7191469

[5] Banoei MM Dinparastisaleh R Zadeh AV Mirsaeidi M. machine-learning-based COVID-19 mortality prediction model and identification of patients at low and high risk of dying. Critical Care. (2021) 25:328. 10.1186/s13054-021-03749-5 34496940 PMC8424411

[6] Douville NJ Douville CB Mentz G Mathis MR Pancaro C Tremper KK. Clinically applicable approach for predicting mechanical ventilation in patients with COVID-19. Br J Anaesth. (2021) 126:578–89. 10.1016/j.bja.2020.11.034 33454051 PMC7833820

[7] Vickers AJ. Prediction models: revolutionary in principle, but do they do more good than harm? J Clin Oncol. (2011) 29:2951–2. 10.1200/JCO.2011.36.1329 21690474

[8] Wynants L Van Calster B Collins GS Riley RD Heinze G Schuit E. Prediction models for diagnosis and prognosis of COVID-19: systematic review and critical appraisal. BMJ. (2020) 369:m1328. 32265220 10.1136/bmj.m1328 PMC7222643

[9] Huang C Wang Y Li X Ren L Zhao J Hu Y. Clinical features of patients infected with 2019 novel coronavirus in Wuhan, China. Lancet. (2020) 395:497–506. 10.1016/S0140-6736(20)30183-5 31986264 PMC7159299

[10] Chen W Yao M Hu L Zhang Y Zhou Q Ren H. Development and validation of a clinical prediction model to estimate the risk of critical patients with COVID-19. J Med Virol. (2022) 94:1104–14. 10.1002/jmv.27428 34716705 PMC8661796

[11] Wong KC Xiang Y Yin L So HC. Uncovering clinical risk factors and predicting severe COVID-19 cases using UK biobank data: machine learning approach. JMIR Public Health Surv. (2021) 7:e29544. 10.2196/29544 34591027 PMC8485986

[12] Collins GS Reitsma JB Altman DG Moons KG. Transparent reporting of a multivariable prediction model for individual prognosis or diagnosis (tripod): the tripod statement. Ann Intern Med. (2015) 162:55–63. 10.7326/M14-0697 25560714

[13] Watari T Tokuda Y Taniguchi K Shibuya K. Incidence of and Ivermectin Prescription Trends for COVID-19 in Japan. J. Gen. Int. Med. (2022) 5:1–3. 10.1007/s11606-022-07877-8 36323824 PMC9629759

[14] Yehia BR Winegar A Fogel R Fakih M Ottenbacher A Jesser C. Association of race with mortality among patients hospitalized with coronavirus disease 2019 (COVID-19) at 92 us hospitals. JAMA Netw Open. (2020) 3:e2018039. 10.1001/jamanetworkopen.2020.18039 32809033 PMC7435340

[15] Sieurin J Branden G Magnusson C Hergens MP Kosidou K. A population-based cohort study of sex and risk of severe outcomes in COVID-19. Eur J Epidemiol. (2022) 37:1159–69. 10.1007/s10654-022-00919-9 36301399 PMC9607822

[16] Moore BJ White S Washington R Coenen N Elixhauser A. Identifying increased risk of readmission and in-hospital mortality using hospital administrative data: the ahrq elixhauser comorbidity index. Med Care. (2017) 55:698–705. 10.1097/MLR.0000000000000735 28498196

[17] Riley RD Ensor J Snell KIE Harrell FE Jr Martin GP Reitsma JB. Calculating the sample size required for developing a clinical prediction model. BMJ. (2020) 368:m441. 10.1136/bmj.m441 32188600

[18] Ghosh P Karim A Atik ST Afrin S Saifuzzaman M. Expert cancer model using supervised algorithms with a LASSO selection approach. Int. J. Elect. Comp. Eng. (2021) 11:2632–40. 10.11591/ijece.v11i3.pp2632-2640

[19] Yates T Summerfield A Razieh C Banerjee A Chudasama Y Davies MJ. A Population-based cohort study of obesity, ethnicity and COVID-19 mortality in 126 million adults in England. Nat. Commun. (2022) 13:624. 10.1038/s41467-022-28248-1 35110546 PMC8810846

[20] Camacho-Moll ME Ramirez-Daher Z Escobedo-Guajardo BL Davila-Valero JC. Rodriguez-de la Garza BL, Bermudez de Leon M. Abc-goalscl score predicts admission to the intensive care unit and mortality of COVID-19 patients over 60 years of age. BMC Geriatr. (2023) 23:138. 10.1186/s12877-023-03864-8 36899318 PMC9999052

[21] Kandil S Tharwat AI Mohsen SM Eldeeb M Abdallah W Hilal A. Developing a mortality risk prediction model using data of 3663 hospitalized COVID-19 patients: a retrospective cohort study in an egyptian university hospital. BMC Pulm Med. (2023) 23:57. 10.1186/s12890-023-02345-3 36750802 PMC9903412

[22] Agrawal U Bedston S McCowan C Oke J Patterson L Robertson C. Severe COVID-19 outcomes after full vaccination of primary schedule and initial boosters: pooled analysis of national prospective cohort studies of 30 million individuals in England, Northern Ireland, Scotland, and Wales. Lancet. (2022) 400:1305–20. 10.1016/S0140-6736(22)01656-7 36244382 PMC9560746

[23] Liang W Liang H Ou L Chen B Chen A Li C. Development and validation of a clinical risk score to predict the occurrence of critical illness in hospitalized patients with COVID-19. JAMA Intern Med. (2020) 180:1081–9. 10.1001/jamainternmed.2020.2033 32396163 PMC7218676

[24] Williams RD Markus AF Yang C Duarte-Salles T DuVall SL Falconer T. Seek cover: using a disease proxy to rapidly develop and validate a personalized risk calculator for COVID-19 outcomes in an international network. BMC Med Res Methodol. (2022) 22:35. 10.1186/s12874-022-01505-z 35094685 PMC8801189

[25] Clift AK Coupland CAC Keogh RH Diaz-Ordaz K Williamson E Harrison EM. Living risk prediction algorithm (qCOVID) for risk of hospital admission and mortality from coronavirus 19 in adults: national derivation and validation cohort study. BMJ. (2020) 371:m3731. 10.1136/bmj.m3731 33082154 PMC7574532

[26] Knight SR Ho A Pius R Buchan I Carson G Drake TM. Risk stratification of patients admitted to hospital with COVID-19 using the isaric who clinical characterisation protocol: development and validation of the 4c mortality score. BMJ. (2020) 370:m3339. 10.1136/bmj.m3339 32907855 PMC7116472

[27] Marcolino MS Pires MC Ramos LEF Silva RT Oliveira LM Carvalho RLR. Abc(2)-Sph risk score for in-hospital mortality in COVID-19 patients: development, external validation and comparison with other available scores. Int J Infect Dis. (2021) 110:281–308. 10.1093/eurheartj/ehab724.3129 34311100 PMC8302820

[28] Magesh S John D Li WT Li Y Mattingly-App A Jain S. Disparities in COVID-19 outcomes by race, ethnicity, and socioeconomic status: a systematic-review and meta-analysis. JAMA Netw Open. (2021) 4:e2134147. 10.1001/jamanetworkopen.2021.34147 34762110 PMC8586903

[29] Richardson S Martinez J Hirsch JS Cerise J Lesser M Roswell RO. Association of race/ethnicity with mortality in patients hospitalized with COVID-19. PLoS ONE. (2022) 17:e0267505. 10.1371/journal.pone.0267505 35925973 PMC9352026

[30] Jordan RE Adab P Cheng KK. COVID-19: Risk Factors for Severe Disease and Death. BMJ. (2020) 368:m1198. 10.1136/bmj.m1198 32217618

[31] Guan WJ Liang WH Zhao Y Liang HR Chen ZS Li YM. Comorbidity and its impact on 1590 patients with COVID-19 in China: a nationwide analysis. Eur Respir J. (2020) 55:20. 10.1183/13993003.01227-2020 32217650 PMC7098485

[32] Steyerberg EW Steyerberg EW. Overfitting and optimism in prediction models clinical prediction models: a practical approach to development, validation, and updating. Cin Dev. (2019) 14:95–112. 10.1007/978-3-030-16399-0_5

[33] Gude-Sampedro F Fernandez-Merino C Ferreiro L Lado-Baleato O Espasandin-Dominguez J Hervada X. Development and validation of a prognostic model based on comorbidities to predict COVID-19 severity: a population-based study. Int J Epidemiol. (2021) 50:64–74. 10.1093/ije/dyaa209 33349845 PMC7799114

[34] Subramanian J Simon R. Overfitting in prediction models–is it a problem only in high dimensions? Contemp Clin Trials. (2013) 36:636–41. 10.1016/j.cct.2013.06.011 23811117

[35] Sterne JA White IR Carlin JB Spratt M Royston P Kenward MG. Multiple imputation for missing data in epidemiological and clinical research: potential and pitfalls. BMJ. (2009) 5:338. 10.1136/bmj.b2393 19564179 PMC2714692

[36] Jassat W Abdool Karim SS Mudara C Welch R Ozougwu L Groome MJ. Clinical severity of COVID-19 in patients admitted to hospital during the omicron wave in south africa: a retrospective observational study. The Lancet Global health. (2022) 10:e961–e9. 10.1016/S2214-109X(22)00114-0 35597249 PMC9116895

[37] Castelli JM Rearte A Olszevicki S Voto C Del Valle Juarez M Pesce M. Effectiveness of Mrna-1273, Bnt162b2, and Bbibp-Corv vaccines against infection and mortality in children in Argentina, during predominance of delta and omicron COVID-19 variants: test negative, case-control study. BMJ. (2022) 379:e073070. 10.1136/bmj-2022-073070 36450402 PMC9709697

[38] Seyler L Van Nedervelde E De Cock D Mann C Pien K Allard SD. Surfing the waves: differences in hospitalised COVID-19 patients across 4 variant waves in a belgian university hospital. Viruses. (2023) 15:618. 10.3390/v15030618 36992327 PMC10057609

